# Correlation Between Physical Activity and Learning Concentration, Self-Management, and Interpersonal Skills Among Korean Adolescents

**DOI:** 10.3390/children11111328

**Published:** 2024-10-30

**Authors:** Jeonga Kwon, Su-Yeon Roh, Daekeun Kwon

**Affiliations:** 1 Department of Elementary Education, College of First, Korea National University of Education, Cheongju 28173, Republic of Korea; gilddong1234555@knue.ac.kr; 2 Department of Exercise Rehabilitation, Gachon University, Incheon 21936, Republic of Korea; dr.rohpilates@gachon.ac.kr; 3 Institute of Sports Health Science, Sunmoon University, Asan 31460, Republic of Korea

**Keywords:** adolescents, learning concentration, interpersonal skills, physical activity, self-management competency

## Abstract

This study aimed to investigate the association between physical activity participation and key developmental competencies—learning concentration, self-management, and interpersonal skills—among Korean adolescents. Data were drawn from the 2021 Study on the State of Play Culture of Children and Adolescents and Measures to Support Growth, conducted by the Korea Adolescent Policy Institute. The study participants included elementary, middle, and high school students in Korea, consisting of 1507 males (53.3%) and 1322 females (46.7%), totaling 2829. We analyzed the data using frequency distribution, chi-square test, and multivariate logistic regression. The results revealed a significant sex difference in the duration of physical activity, with boys spending more time engaging in physical activity than girls. In addition, time spent on physical activities decreased as the school grade level increased. Notably, the more time Korean adolescents spent engaging in daily physical activity, the higher they rated their learning concentration, self-management, and interpersonal skills. These findings suggest the need to promote greater physical activity among adolescents—particularly among girls—which can help improve learning concentration, self-management, and interpersonal skills.

## 1. Introduction

Adolescents spend a significant amount of time studying. Although the benefits of physical activity are widely recognized, the time students dedicate to schoolwork reduces the time available for exercise according to the recommended daily guidelines [1,2,3]. Research shows that physical activity in adolescent affects their mental and emotional states, including cognitive function, academic performance, quality of life, and depression, as well as their physical health, including the occurrence of conditions such as cardiovascular disease, diabetes, cancer, hypertension, obesity, and osteoporosis [4,5,6,7]. Therefore, more attention needs to address the imbalance between time spent on academic study and time allocated for physical activity [4,5,6,7].

Adolescence is an important developmental period during which children mature intellectually, physically, and socially, with heightened brain plasticity and neurocognitive development, particularly in areas such as the prefrontal cortex. This plasticity allows the adolescent brain to respond to external stimuli positively, providing the opportunity for the development of talent and lifelong interests [8]. Research has demonstrated improvements in cognitive performance among children of women who exercised regularly during pregnancy and in individuals who were physically active during childhood and adolescence [9]. This suggests a close relationship between physical activity and cognitive ability. Among cognitive functions, concentration has received the most attention in the adolescent stage [10]. Concentration, defined as the ability to maintain a high level of attention accurately and on a specific stimulus, is considered necessary for success in a wide range of tasks [11]. In other words, adolescent physical activity may be closely related to improvements in their cognitive ability. However, studies conducted in Korea that examined the relationship between physical activity and concentration have yielded mixed results. For example, Kim et al. [12] found no statistically significant relationship between participation in a 12-week basketball program and concentration levels among Korean middle school students. In contrast, Kim [13] observed that concentration levels in Korean middle school students who participated in physical activity differed significantly from that of adolescents who did not participate in physical activity. Thus, a deeper understanding of the relationship between learning concentration and physical activity is needed, considering its importance during adolescence.

Self-management is the process of controlling one’s behavior to pursue and achieve a specific goal. It is a driving force in personal growth, as it encourages behavioral changes aligned with one’s goals and contributes to developing a sense of responsibility and autonomy [14]. Self-management is the rigorous implementation of actions to achieve the goal and is a common characteristic of those who are successful [15]. Research indicates that adolescents can become independent of their parents as they transition into adulthood through successful self-management practices that include managing their physical health, participating in learning, and managing their allowance (pocket money). To achieve this, adolescents need to develop self-management competency. Given that physical activity has a positive impact on various aspects of adolescent development, it may also support their self-management competency. In this regard, examining the relationship between physical activity and self-management competency is essential. Several studies in South Korea have examined the relationship between physical activity and self-management competency; however, these studies only focused on students majoring in physical activity or college-aged adults [15,16]. Given the growing need for self-management competency, there is a need for studies of general adolescents rather than those who specialize in sports.

Adolescents are encouraged to develop competencies that will help them function as healthy members of society by cultivating the basic skills needed to be democratic citizens [17]. In addition to these civic skills, young people need to cultivate interpersonal skills, which involve psychological modalities that shape how individuals think, act toward others, and respond in their relationships with others [18]. Interpersonal skills help adolescents adapt, build self-awareness, and form an individual identity through effective communication and awareness of others [18]. Among adolescents, interpersonal competency has a positive effect on their sense of well-being and supports their healthy development [19]. Studies in different countries that have examined the association between participation in physical activity and interpersonal competency have yielded different results [20,21,22]. For example, Wilson et al. [22], found no statistically significant association between physical activity and interpersonal competency. Meanwhile, Lin et al. [20] reported a significant association between the two variables. In addition, Kim [23] analyzed the relationship between sports activities and interpersonal competency among Korean male middle school students and found a positive effect on the improvement of interpersonal competency in sports activities of middle school boys. However, as Kim [23] noted, the study was limited to Korean male middle school students, highlighting the need to further explore the relationship between the time spent participating in physical activity and interpersonal competency among Korean adolescents.

Factors such as learning concentration, self-management, and interpersonal skills are necessary for young people to develop competencies in democratic citizenship. However, it remains unclear whether physical activity can effectively enhance these skills—particularly concentration, self-management, and interpersonal skills—among Korean adolescents [12,13]. For example, the effect of physical activity on learning concentration among Korean adolescents varies across studies, highlighting the need for research on a large sample [12,13]. Thus, investigating the relationships between the time spent in physical activity, and the development of learning concentration, self-management, and interpersonal skills is warranted. The hypotheses of this study are as follows. (1) participation in physical activity is positively associated with learning concentration among Korean adolescents; (2) participation in physical activity is strongly associated with self-management among Korean adolescents; and (3), Korean adolescents’ participation in physical activity is positively correlated with interpersonal skills.

This study aimed to investigate the relationship between participation in physical activity and three key developmental factors—learning concentration, self-management, and interpersonal skills—among Korean adolescents. Physical activity, along with these competencies, are all important for adolescents’ growth and development. In particular, self-management is an essential competency for future societal participation. During the survey conducted by the Korea Adolescent Policy Institute, students were provided with questionnaires and assistance to clarify any questions they had. While existing research on this topic is limited in Korea, it is gaining attention internationally. We believe that the results of our study can serve as basic data for the further development of policies that promote adolescent physical activity in Korea.

## 2. Materials and Methods

### 2.1. Design and Study Population

We collected data from a 2021 survey on the actual state of play culture and growth support measures for children and adolescents by the Korea Adolescent Policy Institute. The survey included questions on adolescent psychological well-being, propensity to engage in play activities, environment for play activities, and desire for play activities. It included information on interpersonal relationships, learning attitudes, self-management skills, time spent on physical activities and hobbies, and support for play activities. The Korea Adolescent Policy Institute surveyed a total of 2992 students, including 1006 elementary school students, 978 middle school students, and 999 high school students, from May to August in 2021. The study includes data from 2829 participants, excluding 163 participants who provided incomplete responses. The purpose and content of the survey were explained to the students who agreed to participate with consent from a parent or a guardian. Each participant received a questionnaire and filled it out independently, with researchers remaining available to clarify any questions as needed. The survey responses were uploaded to a secure homepage using ID numbers to protect participant anonymity.

For our study, we obtained the raw dataset of this survey following approval (www.nypi.re.kr/archive/mps, accessed on 30 June 2024). Since the dataset did not include any identifier information, ethical approval was not required. Our study was conducted according to the principles outlined in the Declaration of Helsinki. All participants and their guardians were informed about the study’s purpose and voluntarily signed an informed consent form.

### 2.2. Measures

#### 2.2.1. Independent Variable

The independent variable in our study was time spent in physical activity. Time spent in physical activity was measured by the questions to the questions, “How much physical activity do you do on average per day?” The options ranged from none to more than 120 min. Responses were recorded on the following scale: 1 point (no time), 2 points (1–30 min), 3 points (30–60 min), 4 points (60–90 min), 5 points (90–120 min), and 6 points (>120 min).

#### 2.2.2. Dependent Variables

The dependent variables were learning concentration, self-management, and interpersonal skills. Learning concentration was measured by the question, “Do you pay attention in class?” Self-management competency was measured by the question, “Do you manage your personal allowance and make plans to execute the tasks assigned to you, and take the initiative in your work?” Interpersonal skills were assessed by asking, “Do you often hang out with different friends and form good relationships?” Responses to each were measured on a five-point Likert scale: not at all (1 point), no (2 points), normal (3 points), yes (4 points), and very much so (5 points). The responses were used verbatim without modification. The reference category for each dependent variable was set to “not at all”.

#### 2.2.3. Covariate Variables

The covariate variables included sex and school level. Sex was self-reported as either male or female. School grade level was categorized into elementary, middle, and high school. The responses were used without any modifications.

### 2.3. Data Analysis

Data analysis was conducted using SPSS for Windows (version 23.0; IBM Corp., Armonk, NY, USA). First, we conducted a frequency analysis to describe student characteristics. Second, a chi-square analysis was conducted to identify differences in characteristics in terms of time spent in physical activity. Third, we performed multivariate logistic regression to examine the association between time spent in physical activity and learning concentration, self-management, and interpersonal skills. We present the odds ratios (OR), 95% confidence intervals (CI), and *p*-values, with statistical significance set at *p* < 0.05.

## 3. Results

### 3.1. Characteristics of the Students

The demographic characteristics of the students are presented in Table 1. Of the 2829 students, 1507 were male (53.3%) and 1322 were female (46.7%). Among the 2829 participants, 938 (33.2%) were elementary school students, 978 (34.5%) were middle school students, and 913 (32.3%) were high school students. Regarding time spent in physical activity, responses were as follows: 577 students (20.4%) reported “no time”; 712: 1–30 min (25.2%); 822: 30–60 min (29.1%); 238: 60–90 min (8.4%); 263: 90–120 min (9.2%); and 217: >120 min (7.7%). Nearly 40% rated their learning concentration as “normal” (1104) (39.0%); moreover, 923 (32.6%) responded “yes” for self-management competency; and over 40% answered “yes” to the question about socializing with different friends, which measured their interpersonal skills (1159).

### 3.2. Differences Based on Time Spent in Physical Activity

Table 2 presents the results of the differences in the student characteristics based on the time spent in physical activity. We found significant differences in terms of sex (χ^2^ = 92.649, *p* < 0.001), educational level (χ^2^ = 207.774, *p* < 0.001), concentration on learning (χ^2^ = 51.910, *p* < 0.001), self-management (χ^2^ = 80.844, *p* < 0.001), and interpersonal skills (χ^2^ = 85.551, *p* < 0.001).

### 3.3. Association Between Time Spent in Physical Activity and Learning Concentration

The results of multivariate logistic regression analysis examining the association between time spent in physical activity and learning concentration with the ORs and 95% CIs, are presented in Table 3. The reference category for learning concentration was set to “not at all”. The OR for those responding “normal” was 2.069 (range: 1.071–3.997; *p* = 0.030) among those engaging in 30–60 min of activity, and the OR for those responding “yes” was 2.302 (range 1.182–4.485; *p* = 0.014) for that same level of time spent in physical activity. These findings suggest that the more time spent engaging in physical activity, the better the learning concentration.

### 3.4. Association Between Time Spent in Physical Activity and Self-Management Competency

The results of the multivariate logistic regression analysis examining the association between time spent in physical activity and self-management competency (with ORs and 95% CIs) are presented in Table 4. The reference category for self-management competency was set to “not at all”. Among those who engaged in 1–30 min of physical activity, the OR for those who responded “yes” was 1.964 (95% CI: 1.158–3.329; *p* = 0.012). For participants who engaged in more than 120 min of physical activity, the OR for a response of “yes” was 2.568 (95% CI: 1.118–5.899; *p* = 0.026) among those engaging in more than 120 min of physical activity, whereas the OR for those responding “very much so” was 2.770 (95% CI: 1.149–6.679; *p* = 0.023) among those who spent more than 120 min engaging in physical activity. These findings suggest that the more time adolescents spend in physical activity, the higher they rate their self-management competency.

### 3.5. Association Between Time Spent in Physical Activity and Interpersonal Skills

The results of the multivariate logistic regression analysis for the association between the time spent on physical activity and interpersonal skills (with ORs and 95% CIs) are presented in Table 5. The reference category interpersonal competency was set to “not at all”. The OR among those responding “normal” was 3.282 (95% CI: 1.083–9.946; *p* = 0.036) in those engaging in 1–30 min of physical activity and 3.431 (95% CI: 1.112–10.584; *p* = 0.032) in those engaging in 30–60 min of physical activity; the OR among those responding “yes” was 3.839 (95% CI: 1.270–11.606; *p* = 0.017) in those engaging in 1–30 min of physical activity and 5.123 (95% CI: 1.667–15.742; *p* = 0.004) in those engaged in 30–60 min of physical activity. The OR for those responding “very much so” was 4.077 (95% CI: 1.332–12.481; *p* = 0.014) in those engaging in 1–30 min of physical activity; 6.733 (95% CI: 2.168–20.910; *p* = 0.001) in those engaging in 30–60 min of activity; 4.313 (95% CI: 1.095–16.994; *p* = 0.037) in those engaging in 60–90 min in physical activity; 4.076 (95% CI: 1.039–15.992; *p* = 0.044) in those engaging in 90–120 min of physical activity; and 5.818 (95% CI: 1.179–28.719; *p* = 0.031) in those engaging in more than 120 min of physical activity. These findings suggest that adolescents who spent more time engaging in physical activity reported higher levels of confidence in their interpersonal skills.

## 4. Discussion

In our study, we investigated the relationship between time spent in physical activity and learning concentration, self-management, and interpersonal skills among Korean adolescents. Our findings revealed that male students spent more time engaging in physical activity than female students. In addition, we found that the participation rate among adolescents decreased as their school level increased. Notably, the more Korean adolescents participated in physical activity, the higher their self-reported learning concentration, self-management competency, and interpersonal skills.

First, in terms of the trend showing that Korean adolescent boys participated more than adolescent girls in physical activity, Guthold et al. [24], who studied trends in adolescent physical activity in 298 countries, also found that female adolescents were less likely than their male counterparts to engage in physical activity, suggesting the need for greater encouragement of female participation in school. In particular, adolescent girls not only showed lower levels of physical activity but also this level decreased even further as they aged. A systematic review of adolescent girls found a negative correlation between physical activity participation and age [25]. Factors that hinder girls’ participation in physical activity include a lack of friends to participate with, disinterest in physical activity, absence of facilities in the school environment for showering and changing clothes, and societal perception of femininity [26]. These results suggest a critical need to promote increased participation in physical activity among female adolescent by enhancing their motivation through appealing physical activities that they can enjoy with their peers. Hopkins et al. [27] conducted a systematic review of factors associated with female sports participation, showing that the factors influencing girls’ participation in sports were diverse, including personal, peer, family, socioeconomic, environmental, and other factors [27]. To identify ways to reduce the barriers to participation in physical activity among female adolescents in Korea, we need to consider the factors and constraints identified in previous studies [26,27].

Second, our results showed that the participation rate of Korean adolescents in physical activity decreased as they advanced through school levels. This finding has been confirmed in several previous studies [28,29,30]. Shull et al. [29] confirmed that physical activity declined during the transition from seventh to ninth grade. Pate et al. [30] examined the changes in physical activity participation among girls in grades 8–12, showing that girls’ participation in physical activity decreased from 45.4% in the eighth grade to 34.1% in the twelfth grade, consistent with our findings. The reason for the reduced participation of Korean adolescents in physical activity as they progress through grades, is probably the increasing need to spend more time studying for college entrance exams. Korean adolescents face significant pressure from entrance exam competitions, resulting in less time for regular physical activity [31].

Meyer et al. [32] emphasized that school physical education serves as the primary source of physical activity for adolescents. This indicates that adolescents engage in physical activity predominantly within schools, while their participation in physical activity outside of school is limited. However, the results of our study suggest that the Korean physical education curriculum has not been properly implemented [33]. Therefore, there is a need to create opportunities and foster an environment that encourages Korean adolescents to participate in physical activity regularly.

Third, we found that the more Korean adolescents participated in physical activity, the better their learning concentration. Putra et al. [34] explored the association between physical activity participation and improved concentration among Indonesian high school students. Their results showed a significant relationship between physical activity and increased learning concentration, which is consistent with our findings in Korea. Similarly, Silva et al. [35] found that adolescents with attention deficit hyperactivity disorder (ADHD) who participated in physical activity had improved learning concentration compared with those who did not, further supporting our findings. This suggests that participation in physical activity helps adolescents with ADHD improve their concentration on learning and overall school performance. In addition, Polevoy et al. [36] found that physical activity was significantly associated with improved performance and concentration in class. For example, when students aged 8–10 years participated in a set of exercises known as a “physical education minute” during lessons, their concentration improved significantly [36]. Yanti et al. [37] explained that low levels of physical activity result in reduced oxygen transport, which decreases the energy produced by the body’s metabolism [37]. When metabolic processes are suboptimal, the energy required for the learning process is impaired [37]. Low hemoglobin levels in adolescents reduce learning ability, concentration, and endurance [37]. Therefore, the implication is that participation in physical activity increases learning concentration among adolescents by increasing their oxygen and hemoglobin levels.

Fourth, greater participation in physical activity among Korean adolescents was associated with improved self-management. Trost et al. [38] explored the relationship between participation in physical activity and self-management competency among high school students in the U.S. Their findings showed that higher physical activity was associated with self-management competency, consistent with our findings in Korea. Baeck et al. [15] examined differences in self-management levels among college students who did and did not participate in physical activity. Their results showed that college students who participated in physical activity had higher self-management competency than those who did not [15], which partially aligns with our findings [15]. Active participation in physical activity is positively correlated with self-management competency, as concentration and a positive psychological state developed through physical activity enhance self-management skills [39,40].

Fifth, greater participation in physical activity among Korean adolescents was associated with improved interpersonal skills. Lin et al. [20] examined the relationship between physical activity and interpersonal relationships in a study of 542 Chinese adolescents. Their findings confirmed that physical activity positively influences adolescents’ interpersonal relationships, consistent with our findings. Similarly, Ortega–Gómez et al. [21] reported that among Spanish adolescents, higher levels of lower-limb muscle strength, cardiorespiratory fitness, and speed-agility could contribute to better interpersonal relations. Increased physical activity improves interpersonal competency as it encourages interactions between those who have never met and can be an effective way to form positive interpersonal relationships [41]. During physical activity, individuals need to understand their position and take on the responsibility and obligation to coordinate with the others involved, which is essential for improving interpersonal competence [42]. Through cooperation and competition, communication increases, promoting the establishment and further development of these interpersonal relationships [42].

### 4.1. Practical Implications

Our results showed that adolescent girls were less likely than boys to participate in physical activity, and high school students participated in physical activities daily less than elementary school students. These findings highlight the need for strategies to revitalize adolescent physical activity in Korea.

First, concrete actions are needed to promote physical activity among female adolescents. In the United States, girls’ participation in physical activity significantly increased following the enactment of Title IX of the Education Amendment of 1972, which prohibits sex discrimination in school programs and activities [43]. This legislation positively impacted stereotypes about female participation in sports, boosted economic growth in women’s sports, promoted higher education through the management of universities and professional teams, and challenged the perception of women as physically weaker [43]. In Korea, various policies have been proposed that support female adolescent physical activity as one of four key tasks in Korea’s “Plan for the Promotion of Physical Education in Elementary and Secondary Schools” [44]. In a positive step forward, the School Sports Promotion Act proposed revitalization of girls’ sports activities and provided a practical legal basis for further encouraging girls’ sports [45]. Subsequently, specific proposals have been implemented, such as the establishment of classes for girls’ preferred sports, the revitalization of sports clubs for girls’ desired sports, the expansion of indoor gyms and changing rooms, and the prioritization of girls’ preferred sports when equipping physical education departments [45]. However, despite these efforts and 8 years since the legal foundation was set, girls’ participation in physical activities remains a persistent problem in schools. This suggests that, although legislation supporting girls’ physical activity exists, implementation in Korean schools remains limited. Therefore, concrete measures are needed to revitalize physical activity among Korean female adolescents. If physical activity programs for girls are developed and disseminated at the national level, the feasibility of implementing them in schools will improve. In the United States, for example, the “Girls on the Run” program was developed, and in Scotland, the “Fit for Girls” program was launched to promote girls’ physical activity [46,47]. Such efforts are preferable as they involve the government in supporting the development and implementation of detailed programs for girls’ physical activity from planning to implementation. Korea also needs to develop and popularize specific detailed programs to promote the physical activity of female adolescents under the leadership of the government.

Second, there is a need to improve the physical education curriculum in Korean high schools. In Korea, the curriculum is compulsory until tenth grade; however, from eleventh grade onwards, it is optional. By eleventh grade, high school students are focused on college entrance examinations, and physical education and elective subjects have limited impact on these examinations, lessening student interest in physical activities. In the 2015 revision of the physical education curriculum, the number of elective courses in physical education increased from three to four, as the number of units of physical education was reorganized into six semesters, with a total of 10 units or more [48]. However, although the number of courses was increased, most were chosen by student-athletes, with a limit to the use of elective courses by non-student athletes attending general high schools [49]. In addition, elective courses in physical education are designed to impact basic and practical skills. Yet critics have noted that the curriculum remains overly focused on theory-oriented subjects, which lack sufficient connection with practical skills-building [49]. This suggests that physical education in school may not be effectively serving as the primary means of promoting physical activity among adolescents [32].

Recently, Korea’s 2015 Revised Physical Education Curriculum was updated to the 2022 Revised Physical Education Curriculum, which introduce high school credit system. The high school credit system provides students with autonomous choices by offering courses based on student demand and systematically reflects process-based assessments to evaluate course completion and graduation according to whether students meet achievement standards [50]. Under the high school credit system, set to take effect in 2025, the required number of credits has been reduced from 204 to 192. However, teachers in the field are concerned that subjects unrelated to entrance exams may be excluded from high school credits centered on these exams [51]. In other words, considering Korea’s entrance examination system, high school students are less likely to choose physical education subjects that lack connection with entrance exams. While it is essential to provide high school students with the freedom to choose their physical activity involvement, it is equally important to ensure they understand the value of physical activity and lay the foundation for managing and maintaining their health through participation in physical activity in a balanced manner between the theory and practice. To support this, expanding and ensuring the required 10 designated credits for physical education is necessary.

### 4.2. Limitations

However, this study has certain limitations that warrant attention. First, as a secondary study, we were limited in our ability to assess temporal antecedents or causal relationships. Our data were derived from the 2021 Study on the State of Play Culture of Children and Adolescents and Measures to Support Growth. Because we used their responses on learning concentration, self-management, and interpersonal skills, we could not establish causality between these variables. Second, the time spent engaging in physical activity was not measured accurately, as participants self-reported their time spent engaging in physical activity based on personal estimations. Thus, self-reporting bias and common method bias could have influenced our results. Future research could address these limitations through longitudinal study designs and a mixed-method approach, incorporating both qualitative interviews and quantitative analysis. Third, we did not examine the association between variables by time of physical activity, sex, and age, which has been acknowledged as a limitation of this study. Fourth, the questionnaires used were not directly validated by the researchers, as the study utilized secondary data from the Korea Adolescent Policy Institute. However, the Korea Adolescent Policy Institute developed the questionnaire in consultation with experts, ensuring validity and reliability to some extent.

## 5. Conclusions

Our findings revealed that adolescent boys in Korea participated in physical activity significantly more than girls, and the participation rate decreased as the school level increased. Notably, greater engagement in physical activity among adolescents was associated with higher ratings of learning concentration, self-management, and interpersonal skills. Based on these positive effects of physical activity on academic performance, it is essential to continue promoting physical activity among Korean adolescents, both in and out of school. At the national level, in particular, detailed programs should be developed to encourage and promote physical activity among girls, empowering them to take the initiative in participating in physical activity both within and outside of school settings. Additionally, increasing the required number of credits for physical education classes in high school would allow students more opportunities to engage in physical activity through classes.

## Figures and Tables

**Table 1:** Student characteristics (n = 2829).

Characteristic	Category	n (%)
Sex	Male	1507 (53.3%)
	Female	1322 (46.7%)
School grade level	Elementary school	938 (33.2%)
	Middle school	978 (34.5%)
	High school	913 (32.3%)
Time spent in physical activity	>120 min	217 (7.7%)
	90–120 min	263 (9.2%)
	60–90 min	238 (8.4%)
	30–60 min	822 (29.1%)
	1–30 min	712 (25.2%)
	No time	577 (20.4%)
Concentration on learning	Not at all	77 (2.7%)
	No	403 (14.2%)
	Normal	1104 (39.0%)
	Yes	945 (33.4%)
	Very much so	300 (10.6%)
Self-management	Not at all	95 (3.4%)
	No	307 (10.9%)
	Normal	862 (30.5%)
	Yes	923 (32.6%)
	Very much so	642 (22.7%)
Interpersonal skills	Not at all	29 (1.0%)
	No	131 (4.6%)
	Normal	652 (23.0%)
	Yes	1159 (41.0%)
	Very much so	858 (30.3%)

**Table 2:** Differences in the student characteristics based on time spent in physical activity (n = 2829).

Characteristic	Category	Time Spent in Physical Activity						χ^2^ (*p*)
		>120 min	90–120 min	60–90 min	30–60 min	1–30 min	No Time	
Sex	Male	156 (71.9%)	171 (65.0%)	139 (58.4%)	465 (56.6%)	335 (47.1%)	241 (41.8%)	92.649 (<0.001 ***)
	Female	61 (28.1%)	92 (35.0%)	99 (41.6%)	357 (43.4%)	377 (52.9%)	336 (58.2%)	
School grade level	Elementary school	99 (45.6%)	92 (35.0%)	122 (51.3%)	322 (39.2%)	222 (31.2%)	81 (14.0%)	207.774 (<0.001 ***)
	Middle school	66 (30.4%)	93 (35.4%)	81 (34.0%)	282 (34.3%)	254 (35.7%)	202 (35.0%)	
	High school	52 (24.0%)	78 (29.7%)	35 (14.7%)	218 (26.5%)	236 (33.1%)	294 (51.0%)	
Concentration on learning	Not at all	4 (1.8%)	5 (1.9%)	4 (1.7%)	17 (2.1%)	19 (2.7%)	28 (4.9%)	51.910 (<0.001 ***)
	No	32 (14.7%)	34 (12.9%)	23 (9.7%)	109 (13.3%)	95 (13.3%)	110 (19.1%)	
	Normal	89 (41.0%)	119 (45.2%)	100 (42.0%)	298 (36.3%)	279 (39.2%)	219 (38.0%)	
	Yes	61 (28.1%)	79 (30.0%)	89 (37.4%)	313 (38.1%)	232 (32.6%)	171 (29.6%)	
	Very much so	31 (14.3%)	26 (9.9%)	22 (9.2%)	85 (10.3%)	87 (12.2%)	49 (8.5%)	
Self-management competency	Not at all	9 (4.1%)	13 (4.9%)	9 (3.8%)	59 (7.2%)	37 (5.2%)	40 (6.9%)	80.844 (<0.001 ***)
	No	18 (8.3%)	38 (14.4%)	37 (15.5%)	145 (17.6%)	120 (16.9%)	145 (25.1%)	
	Normal	75 (34.6%)	84 (31.9%)	84 (35.3%)	278 (33.8%)	249 (35.0%)	216 (37.4%)	
	Yes	65 (30.0%)	85 (32.3%)	60 (25.2%)	221 (26.9%)	213 (29.9%)	114 (19.8%)	
	Very much so	50 (23.0%)	43 (16.3%)	48 (20.2%)	119 (14.5%)	93 (13.1%)	62 (10.7%)	
Interpersonal competency	Not at all	2 (0.9%)	3 (1.1%)	3 (1.3%)	5 (0.6%)	5 (0.7%)	11 (1.9%)	85.551 (<0.001 ***)
	No	9 (4.1%)	8 (3.0%)	10 (4.2%)	32 (3.9%)	33 (4.6%)	39 (6.8%)	
	Normal	37 (17.1%)	42 (16.0%)	38 (16.0%)	178 (21.7%)	187 (26.3%)	170 (29.5%)	
	Yes	79 (36.4%)	110 (41.8%)	93 (39.1%)	344 (41.8%)	299 (42.0%)	234 (40.6%)	
	Very much so	90 (41.5%)	100 (38.0%)	94 (39.5%)	263 (32.0%)	188 (26.4%)	123 (21.3%)	

Note: *** *p* < 0.001, assessed using chi-squared analyses.

**Table 3:** Association between time spent in physical activity and learning concentration.

Variable		Learning Concentration (Odds Ratio (95% Confidence Interval))				
		Not at All (n = 77)	No (n = 403)	Normal (n = 1104)	Yes (n = 945)	Very Much So (n = 300)
Time spent in physical activity	>120 min	1.000	2.157 (0.679–6.851) *p* = 0.192	2.158 (0.780–6.573) *p* = 0.176	1.347 (0.434–4.181) *p* = 0.606	1.595 (0.479–5.304) *p* = 0.447
	90–120 min	1.000	1.854 (0.648–5.303) *p* = 0.250	2.611 (0.957–7.125) *p* = 0.061	1.685 (0.607–4.679) *p* = 0.317	1.394 (0.459–4.234) *p* = 0.558
	60–90 min	1.000	1.531 (0.471–4.979) *p* = 0.479	2.460 (0.808–7.493) *p* = 0.113	2.059 (0.669–6.335) *p* = 0.208	1.321 (0.390–4.483) *p* = 0.655
	30–60 min	1.000	1.796 (0.902–3.576) *p* = 0.095	2.069 (1.071–3.997) *p* = 0.030 *	2.302 (1.182–4.485) *p* = 0.014 *	1.811 (0.860–3.816) *p* = 0.118
	1–30 min	1.000	1.289 (0.667–2.491) *p* = 0.449	1.618 (0.866–3.024) *p* = 0.131	1.484 (0.785–2.805) *p* = 0.225	1.697 (0.833–3.458) *p* = 0.145

Note: * *p* < 0.05, assessed using multivariate logistic regression analysis adjusted for sex and school grade.

**Table 4:** Association between time spent in physical activity and self-management.

Variable		Self-Management (Odds Ratio [95% Confidence Interval])				
		Not at All (n = 95)	No (n = 307)	Normal (n = 862)	Yes (n = 923)	Very Much So (n = 642)
Time spent in physical activity	>120 min	1.000	0.575 (0.233–1.420) *p* = 0.230	1.465 (0.652–3.293) *p* = 0.355	2.568 (1.118–5.899) *p* = 0.026 *	2.770 (1.149–6.679) *p* = 0.023 *
	90–120 min	1.000	0.767 (0.364–1.617) *p* = 0.486	1.034 (0.510–2.097) *p* = 0.926	2.043 (0.991–4.212) *p* = 0.053	1.515 (0.687–3.341) *p* = 0.304
	60–90 min	1.000	1.154 (0.497–2.681) *p* = 0.739	1.562 (0.698–3.493) *p* = 0.278	2.161 (0.941–4.964) *p* = 0.069	2.277 (0.950–5.461) *p* = 0.065
	30–60 min	1.000	0.649 (0.396–1.064) *p* = 0.086	0.748 (0.465–1.203) *p* = 0.230	1.131 (0.686–1.866) *p* = 0.629	0.854 (0.489–1.492) *p* = 0.579
	1–30 min	1.000	0.931 (0.551–1.574) *p* = 0.790	1.218 (0.735–2.019) *p* = 0.444	1.964 (1.158–3.329) *p* = 0.012 *	1.301 (0.722–2.345) *p* = 0.381

Note: * *p* < 0.05, assessed using multivariate logistic regression analysis adjusted for sex and school grade.

**Table 5:** Association between time spent in physical activity and interpersonal skills.

Variable		Interpersonal Competency (Odds Ratio (95% Confidence Interval))				
		Not at All (n = 29)	No (n = 131)	Normal (n = 652)	Yes (n = 1159)	Very Much So (n = 858)
Time spent in physical activity	>120 min	1.000	1.650 (0.293–9.304) *p* = 0.570	1.975 (0.397–9.817) *p* = 0.405	3.092 (0.631–15.160) *p* = 0.164	5.818 (1.179–28.719) *p* = 0.031 *
	90–120 min	1.000	0.928 (0.200–4.316) *p* = 0.924	1.319 (0.334–5.213) *p* = 0.693	2.563 (0.660–9.951) *p* = 0.174	4.076 (1.039–15.992) *p* = 0.044 *
	60–90 min	1.000	1.301 (0.287–5.896) *p* = 0.733	1.369 (0.344–5.448) *p* = 0.655	2.618 (0.671–10.218) *p* = 0.166	4.313 (1.095–16.994) *p* = 0.037 *
	30–60 min	1.000	2.383 (0.714–7.958) *p* = 0.158	3.431 (1.112–10.584) *p* = 0.032 *	5.123 (1.667–15.742) *p* = 0.004 **	6.733 (2.168–20.910) *p* = 0.001 **
	1–30 min	1.000	2.300 (0.703–7.522) *p* = 0.168	3.282 (1.083–9.946) *p* = 0.036 *	3.839 (1.270–11.606) *p* = 0.017 *	4.077 (1.332–12.481) *p* = 0.014 *

Note: * *p* < 0.05, ** *p* < 0.01, assessed using multivariate logistic regression analysis adjusted for sex and school grade.

## Data Availability

Publicly available datasets were analyzed in this study. These data can be found here: [www.nypi.re.kr/archive/mps, accessed on 30 June 2024].
